# Neutrophil-inspired propulsion in a combined acoustic and magnetic field

**DOI:** 10.1038/s41467-017-00845-5

**Published:** 2017-10-03

**Authors:** Daniel Ahmed, Thierry Baasch, Nicolas Blondel, Nino Läubli, Jürg Dual, Bradley J. Nelson

**Affiliations:** 1 Institute of Robotics and Intelligent Systems, ETH Zurich, Zurich, CH-8092 Switzerland; 2 Institute of Mechanical Systems, ETH Zurich, Zurich, CH-8092 Switzerland

## Abstract

Systems capable of precise motion in the vasculature can offer exciting possibilities for applications in targeted therapeutics and non-invasive surgery. So far, the majority of the work analysed propulsion in a two-dimensional setting with limited controllability near boundaries. Here we show bio-inspired rolling motion by introducing superparamagnetic particles in magnetic and acoustic fields, inspired by a neutrophil rolling on a wall. The particles self-assemble due to dipole–dipole interaction in the presence of a rotating magnetic field. The aggregate migrates towards the wall of the channel due to the radiation force of an acoustic field. By combining both fields, we achieved a rolling-type motion along the boundaries. The use of both acoustic and magnetic fields has matured in clinical settings. The combination of both fields is capable of overcoming the limitations encountered by single actuation techniques. We believe our method will have far-reaching implications in targeted therapeutics.

## Abstract (web summary)

Devising effective swimming and propulsion strategies in microenvironments is attractive for drug delivery applications. Here Ahmed et al. demonstrate a micropropulsion strategy in which a combination of magnetic and acoustic fields is used to assemble and propel colloidal particles along channel walls.

## Introduction

The design parameters of motion at the microscale are distinctly different from their inertia-dominated counterparts^1^. Nature’s microswimmers, such as bacteria^2^, spermatozoa^3, 4^ and volvox^5^, have evolved to overcome swimming limitations in viscous liquids at low Reynolds numbers. These swimmers adhere to Purcell’s scallop theorem, which states that a swimmer undergoing reciprocal motion will achieve no net movement^1, 6^. To date, most synthetic swimmers adopt Purcell’s scallop formulation as a design paradigm for propulsion. Existing microswimmers and nanoswimmers are actuated using different mechanisms such as by chemical fuel^7–14^ or electric fields^15, 16^, light-induced^17–19^ and magnetically powered^20–22^ and they seem promising in a biological or biomedical context^23^. A major objective that we have for artificial microswimmers and nanoswimmers is that they act as drug carriers in the human body, which requires that they have the ability to be propelled in confined boundaries, such as the vasculature. However, limited work exists regarding propulsion within microchannels or in a confined geometry. Both acoustically^24–31^ and magnetically powered microswimmers can potentially be used in the human body. However, the motion of acoustic-based swimmers may be adversely affected when they are confined in channels due to swimmer–wall interactions, i.e. secondary acoustic forces may lead to trapping the swimmers at the walls.

Recently, Tasci et al.^20^ demonstrated magnetic particles that had a rolling-type motion. The described motion was limited to horizontal surfaces because their propulsion mechanism relied on a contact force that inherently depended on the gravitational weight of the particles. Magnetically induced rolling motion of micro-objects in three-dimensional (3D) geometries is challenging because navigation requires the rotational axis of the magnetic field be at a precise angle with respect to the contact boundary. Continuous use of the reaction force will require constant monitoring and precise tracking of the swimmer and corresponding adjustments of the field. In addition, particles that are immersed in liquid experience buoyancy, which acts in opposite directions to the weight, thus minimise the effect of gravity.

We have developed a propulsive mechanism based on combined acoustic and magnetic actuation. To demonstrate rolling motion, micro-sized superparamagnetic particles are injected into the channel, after which a rotating magnetic field is applied. The particles assemble into aggregates due to magnetic interactions. As soon as the acoustic field is activated, the collected particles migrate to the boundary, and a subsequent rolling motion is achieved along the vessel wall. An important feature of our system is the possibility to controlling the normal force, critical for rolling, can be regulated by an acoustic field. We have demonstrated rolling motion along the walls of rectangular and circular microchannels.

## Results

### Particle motion in an acoustic field

High-frequency acoustic fields at moderate levels of pressure are safe, non-invasive and inexpensive, and they are used extensively in clinical diagnostics and therapeutics. Ultrasound also has been utilised in numerous biotechnological and biomedical applications, such as microfluidic-based micromixers^32–34^ and chemical gradient generation^35, 36^, particle/cell manipulation^37–45^, separation of circulating tumour cells^46^, the formation of spheroids^47^, levitation of objects^41^ and high-resolution imaging^48^. Contrast agents, such as microbubbles, have been used to enhance the visibility of vessels in ultrasound-based imaging^48, 49^. Recently, microbubbles in conjunction with ultrasound have been used in the treatment of brain disease^50^ and gastrointestinal pathology^51^. Extensive literature exists regarding the behaviour of microbubbles near boundaries^52–54^ and in vasculatures^55^ in an acoustic field, but little work has been done on the interactions of solid particles near boundaries.

**Fig. 1:**
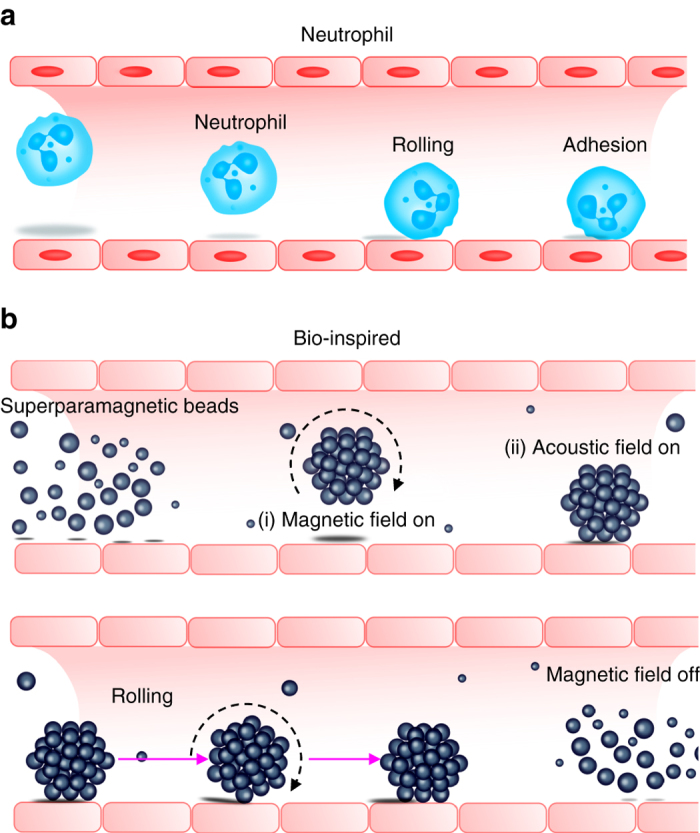
Neutrophil-inspired propulsion based on acoustic and magnetic field actuation. **a** Neutrophil rolls on vasculature before transmigrating into tissue. **b** Superparamagnetic particles aggregate due to dipole–dipole interaction in the presence of a magnetic field (i). The aggregate migrates towards the wall due to the radiation force in an acoustic field (ii). A rolling-type motion along the boundary is achieved by combining both fields. The assembled entities disassociate when the magnetic field is turned off

Inspired by neutrophils rolling on endovascular walls before transmigrating to a disease site, we have developed a propulsive mechanism based on combined acoustic and magnetic actuation (Fig. 1a). Rolling motion is demonstrated by introducing superparamagnetic microparticles into the channel, after which a rotating magnetic field is applied. The particles form aggregates due to dipole–dipole interactions (Fig. 1b). The assembled particles migrate to the boundary in an acoustic field when activated at an appropriate frequency, which resulted in a rolling motion along the vessel wall, as shown in Fig. 1b. When the magnetic field is turned off, the assembled entities disassociate.

**Fig. 2:**
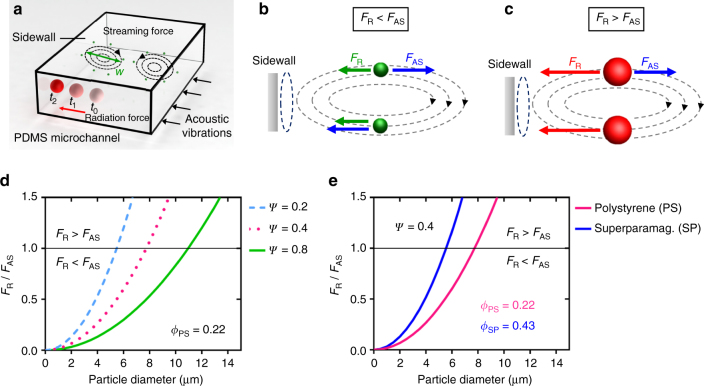
Analysis of particle trapping. **a** A schematic demonstrates the radiation force (*F*_R_) and the sidewall-induced streaming drag (*F*_AS_) that act on different sized particles. Large particles, which are illustrated in *red*, migrate to the channel sidewall by the radiation force. Small particles, which are shown in *green*, follow sidewall-induced streaming. **b** For a particle with a diameter *a* < *a*_c_, the acoustic streaming force dominates. **c** For a particle with a diameter *a* > *a*_c_, the acoustic radiation force dominates. **d** The plot shows the ratio of the acoustic radiation to streaming forces vs. different size polystyrene particle for various geometry factor, *Ψ*. Above the *black line*, *F*_R_ > *F*_AS_, i.e. radiation force dominates and below the *line*, *F*_R_ < *F*_AS_, i.e. streaming dominates. **e** The plot demonstrates the competing forces of the polystyrene (*PS*) and the superparamagnetic particles (*SP*) at *Ψ* ~0.4

We investigated the migration of particles towards the sidewall of a polydimethylsiloxane (PDMS)-based microchannel by introducing particles of different size and material properties. The PDMS material serves as a good acoustic material for developing artificial vasculature model as the acoustic impedance of PDMS is of a similar order of magnitude to that of biomaterials that exist in a human body such as the blood, fat, brain and soft tissue^56^. Several acoustic phenomena, such as the acoustic radiation forces and the streaming-induced drag, contribute to particle motion, as shown in Fig. 2a. The acoustic radiation force, which is a time-averaged force that acts on particles in an inviscid fluid, comprises primary and secondary radiation forces. The primary radiation force arises due to the interactions of particles within the background pressure field, such as in the standing and travelling acoustic waves, and secondary radiation forces arise due to re-scattering of the waves at the neighbouring particles and the sidewalls of the channel. The primary radiation, *F*_R_, force acting on a single isolated particle in an acoustic pressure field can be estimated by the gradient of the Gor’kov potential. The time-averaged force acting on a particle of radius *a*, where *a* is much smaller than the acoustic wavelength, *λ*, in a one-dimensional standing wave of wavenumber *k*_y_ and pressure field *p* = *p*_a_cos(*k*_y_ *y*)cos(*ωt*), can be expressed analytically as^57^:

1 $${F_{\rm{R}}} = 4\pi \phi \left( {\tilde \kappa ,\tilde \rho } \right){k_{\rm{y}}}{a^3}{E_{\rm{a}}}{\rm{sin}}\left( {2{k_{\rm{y}}}y} \right)$$

2 $$\phi \left( {\tilde \kappa ,\tilde \rho } \right) = \frac{1}{3}\left[ {\frac{{5\tilde \rho - 2}}{{2\tilde \rho + 1}} - \tilde \kappa } \right]$$

3 $$\tilde \rho = \frac{{{\rho _{\rm{s}}}}}{{{\rho _0}}},\,\tilde \kappa = \frac{{{\kappa _{\rm{s}}}}}{{{\kappa _0}}}$$

where *ϕ* is the acoustophoretic contrast factor; *ρ*_0_, *ρ*_s_ denote the density of the liquid and particle, respectively; *κ*_0_, *κ*_s_ denote the compressibility of the water and particle, respectively, *p*_a_ denotes the pressure amplitude, *E*_a_ denotes the acoustic energy and *y* determines the position of the particle within the field. The acoustic contrast factor *ϕ* determines the directionality of the radiation force. The *ϕ* for polystyrene and superparamagnetic particles is positive and is calculated to be 0.22 and 0.43, respectively, which suggests that the particles will move to the pressure nodal lines, where pressure nodes may lie outside the microchannel, effectively driving the particles to its boundaries (Supplementary Notes 1 and 2).

We observed very little particle–particle interaction due to secondary acoustic radiation forces in the region away from the sidewalls, which suggests that the primary radiation force dominates particle migration. However, as particles approach the wall, they change direction and accelerate (Supplementary Fig. 3), which indicates a particle–wall interaction. Owing to the impedance mismatch between the water and the PDMS, the acoustic waves are partially reflected at their interface, which leads to secondary interactions between the walls of the channel and the particles^58^. The walls of the channel have been observed to attract the particles at close range. The particle–wall interaction contributes to the migration of particles to the wall, but this interaction is considered to be weak because the impedance mismatch between water and the PDMS is not significant (Supplementary Fig. 3).

Acoustic streaming is a steady bulk flow of fluid that arises due to viscous attenuation of the acoustic waves in the bulk of the fluid and the boundary layers, and it often contributes to the particle motion in an acoustic setup in microfluidics. The streaming flow rises from the channel sidewalls to the centre and is perpendicular to the direction of the long axis of the channel, as shown in Fig. 2a. The magnitude of the streaming velocity *v*_AS_ scales as ${{\psi }}\frac{{p_{\rm{a}}^2}}{{\rho _0^2c_0^3}}$, where *Ψ* is a geometry-dependent factor of the microchannel and *c*_0_ is the speed of sound in the liquid. Since the Reynolds number is low (*R*_e_ < 0.0002), the drag force *F*_AS_ that is induced by streaming can be estimated as follows:

4 $${F_{{\rm{AS}}}} = 6\pi a\eta {\rm{\psi }}\frac{{p_{\rm{a}}^2}}{{\rho _0^2c_0^3}}$$

where *η* is the dynamic viscosity of the liquid. Typically, the amplitude of the Stokes drag acting on spherical particles is on the order of the particle radius, and the acoustic radiation force scales with the particle volume. Thus, for smaller particles, the sidewall-induced streaming drag dominates particle motion until a transition size is reached, beyond which the radiation force becomes more dominant. The trapping mechanism can be understood by considering the ratio of the acoustic radiation forces in a standing wave field and the streaming-induced drag forces that can be expressed by the following relationship:

5 $$\frac{{{F_{\rm{R}}}}}{{{F_{{\rm{AS}}}}}} = \frac{{{\rho _0}{c_0}\phi {k_{\rm{y}}}{a^2}}}{{6\eta {\rm{\psi }}}}$$

The relation is independent of the pressure amplitude, but depends on the particle and fluid properties, channel geometry and the wavenumber. When (*F*_R_)/(*F*_AS_) < 1, the streaming force dominates, as shown in Fig. 2b. When (*F*_R_)/(*F*_AS_) > 1, the acoustic radiation force dominates, as indicated in Fig. 2c. Figure 2d describes the ratio of the competing forces of different size polystyrene particle for various geometry-dependent factor, *Ψ*. The geometry factor of a rectangular microchannel is usually on the order of unity and matches our experimental condition when *Ψ* becomes 0.4, i.e. for a standing wave parallel to a wall^57^. The critical particle diameter, *a*_c_, which determines the transition, can be measured when the acoustic radiation force is exactly balanced by the streaming-induced drag force. The diameter *a*_c_ of polystyrene particles is measured to be ~8 µm, which is determined by the intersection of the magenta plot at (*F*_R_)/(*F*_AS_) = 1 in Fig. 2d. Figure 2e compares the competing forces of the polystyrene and the superparamagnetic particles at *Ψ* ~0.4. As the acoustic contrast force of the superparamagnetic particles is higher than that of the polystyrene particles, the critical particle diameter becomes smaller. The superparamagnetic particle *a*_c_ is estimated to be ~5.5 µm, which is determined by the intersection of the blue plot at (*F*_R_)/(*F*_AS_) = 1 in Fig. 2e.

**Fig. 3:**
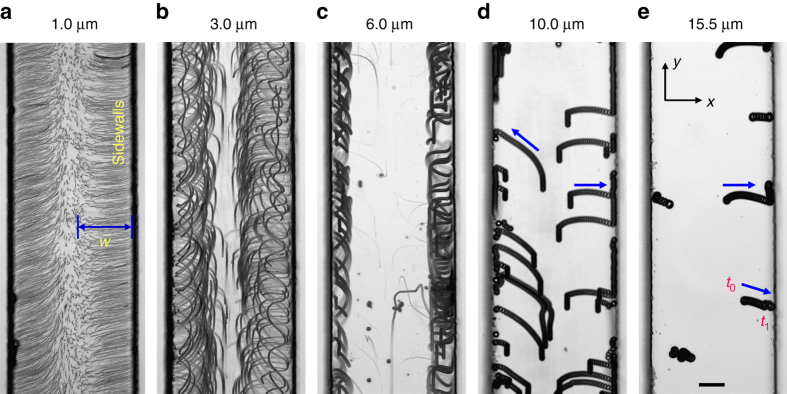
Particle–wall interaction in an acoustic field. Image sequences demonstrate the particle–wall interaction of **a** 1, **b** 3, **c** 6, **d** 10 and **e** 15.5 μm diameter polystyrene particles, respectively, in a microfluidic channel. **a**–**c** Represents the sidewall-induced acoustic streaming developed within the microchannel. The vortex width *w* decreases with increasing particle diameter. **d**, **e** Demonstrates migration and trapping of 10 and 15.5 μm particles, respectively, at the channel sidewalls (see also Supplementary Movie 1). *Blue arrows* represent the particle trajectories, *t*_0_ and *t*_1_ represent the initial and final position, respectively. *Scale bar*, 50 µm

We verified the size dependence of the particle–wall interaction by introducing polystyrene particles of diameter 1, 3, 6, 10 and 15.5 µm, respectively. The 1, 3 and 6 µm particles follow the streamlines of the sidewall-induced streaming, as shown in Fig. 3a–c. The vortex width *w*, which is defined as the distance between the starting and ending vertex points of a vortex, decreases as the diameter of the particles increases as the radiation force becomes prominent. Conversely, the 10 and 15.5 µm particles migrate transversely towards the ±*x* direction and become trapped at the sidewalls, as shown in Fig. 3d and e (see also Supplementary Movie 1). Supplementary Figure 4 demonstrates the trapping of ~6 µm superparamagnetic particles, whereas, similar size polystyrene particles follow the flow streamlines. The experiment results closely agree with the analysis, as shown in Fig. 2d and e (see also Supplementary Movie 2).

### Particle assembly and rolling motion

Inspired by the rolling motion of neutrophils on endovascular walls, we developed a propulsive mechanism based on a combined acoustic and magnetic actuation. The magnitude and the frequency of the magnetic field determine the assembly formation of the superparamagnetic particles. A magnetic field of a few milli-tesla ensures that the dipole–dipole interactions are large enough to attract neighbouring particles. At low rotational frequencies of the magnetic field the superparamagnetic particles tend to form chains. The dipole moments of the particles continuously realign themselves with the direction of the rotating magnetic field. As the Reynolds number is in the Stokes regime, the viscous torque due to drag instantly balances the magnetic torque. The viscous torque^59^ is defined as *τ*_D_ ∝ *ηl* ^3^ *ω*, where *η* is the dynamic viscosity, *l* is the length of the chain and *ω* is the angular frequency of the chain. A longer chain formation of length *l* or a higher angular frequency *ω* increases the viscous torque. The interplay between magnetic and viscous forces determines the self-assembly configuration.

**Fig. 4:**
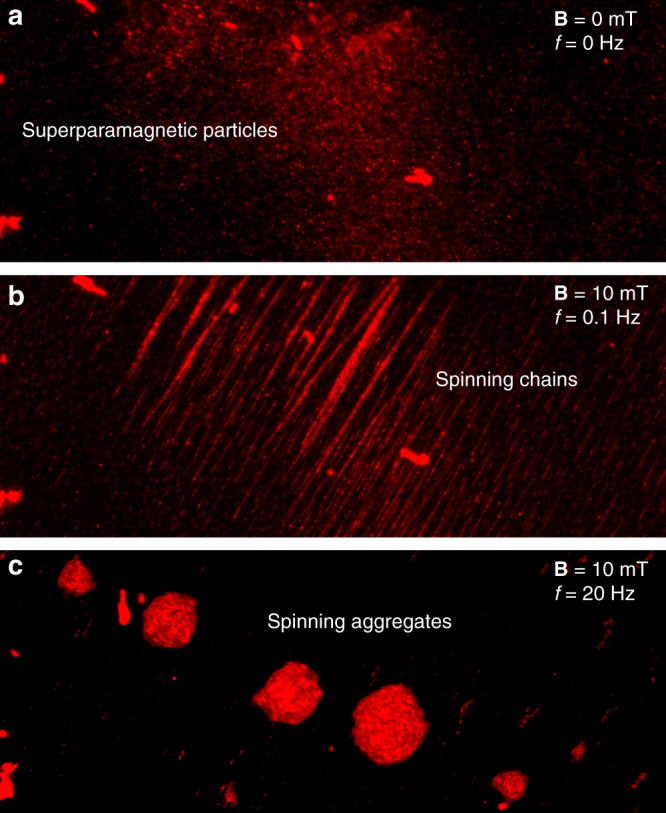
Particle interaction in a rotating magnetic field. **a** 1–2 µm fluorescent superparamagnetic particles are dispersed within the glass slides in the absence of a magnetic field. **b** The superparamagnetic particles self-assemble and form spinning chains at a rotational frequency of 0.1 Hz. **c** The spinning chains fragment into satellites, and eventually the chain transforms into spinning orbits at a higher frequency. The rotation of the spinning aggregates is demonstrated at a rotational frequency of 20 Hz, see also Supplementary Movie 3. *Scale bar*, 100 µm

We sandwiched superparamagnetic particles between glass slides after which a low-strength rotating magnetic field is applied that results in a linear chain formation, as shown in Fig. 4a and b. As the rotational frequency of the magnetic field increases to 20 Hz, the spinning chains begins to fragment into satellites, and eventually the chain transforms into spinning orbits of diameter larger than ~6 µm, which is the critical particle diameter for acoustic trapping at the wall under the current setup, as shown in Fig. 4c (see also Supplementary Movie 3). At low Reynolds number, the time-reversal nature of the spinning orbits ensures negligible net motion. To generate noticeable motion, a swimmer needs to break either the symmetry of its motion or the symmetry of its surrounding geometry.

**Fig. 5:**
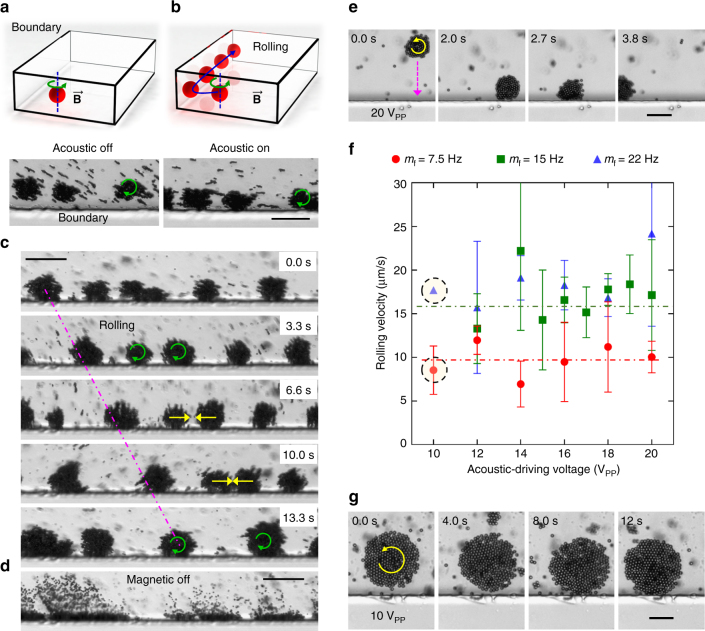
Rolling motion in acoustic and magnetic fields. **a** The 1–2 µm superparamagnetic particles self-assemble and form spinning aggregates rotating in the clockwise direction in the presence of a magnetic field. **b** The aggregate migrates towards the channel wall due to the radiation force of an acoustic field. **c** Image sequence demonstrates the rolling-type propulsion along the sidewall of the microfluidic channel (Supplementary Movie 4). **d** The superparamagnetic particles disassociate as soon as the magnetic field is turned off. *Scale bar* of **b**–**d** is 50 µm. **e** Image sequence demonstrates the counter-clockwise motion of an aggregate and executing rolling when reached the wall under a magnetic field strength and rotation frequency of 20 mT and 20 Hz, respectively, at an acoustic driving voltage of 20 V_PP_ (Supplementary Movie 6). *Scale bar*, 25 µm. **f** The plot of the rolling velocity against acoustic driving voltage at magnetic rotational frequencies (*m*_f_) of 7.5, 15 and 22 Hz, respectively, at 15 mT, of 2.9 µm superparamagnetic particles. Each *data point* represents the average rolling velocity analysed from 3–5 swimmers except data points at 10 V_PP_. The *error bar* represents the standard deviation (s.d.). **g** An aggregate is shown weak to no rolling at low-acoustic driving voltages of 10 V_PP_ under a magnetic field strength and rotation frequency of 15 mT and 7.5 Hz, respectively (Supplementary Movie 7). *Scale bar*, 25 µm

After superparamagnetic particles are injected into the microfluidic channel, multiple spinning aggregates or orbits are formed by tuning the frequency of the magnetic field, as shown in Fig. 5a. The aggregates are shown to rotate in the clockwise direction. When the acoustic field is turned on at an appropriate frequency and amplitude, assembled particles, with a diameter larger than the critical diameter *a*_c_ migrate to the boundaries due to the acoustic radiation force, as shown in Fig. 5b. As a result, the symmetry of the spinning aggregates is broken due to the presence of a boundary, and a subsequent rolling motion from left to right is achieved, as seen in Fig. 5c (see also Supplementary Movie 4). The assembled particles disassociate when the magnetic field is turned off (Supplementary Fig. 5 and Supplementary Movie 5). As the acoustic field is still on, and the diameter of the particles is less than the critical diameter *a*_c_, the particles undergo sidewall streaming as shown in Fig. 5d. Figure 5e shows the counter-clockwise rotation of the aggregates and executing rolling from right to left when reached the wall (see also Supplementary Movie 6). We characterised the rolling behaviour and estimated the minimum power or voltage required by the piezo transducer to establish rolling. Figure 5f demonstrates the rolling velocity for different acoustic voltages at various magnetic rotational frequencies. As we decrease the acoustic voltage, rolling is achieved voltage until a lower acoustic voltage threshold is reached. For example, when the voltage is reduced to 12 or 10 V_PP_, a rolling motion is no longer obtained. At this critical voltage, most of the rollers are unaffected by the acoustic power and the few that reached the wall induced rolling at the wall ﻿transiently. As the contact between a roller and the wall remains weak, the roller also drifts away, as shown in Fig. 5g (see also Supplementary Movie 7). The experiments also suggest that the rolling speed is primarily dependent on the magnetic rotational frequencies, i.e. the average rolling speed increase from ~10 to 17 μm/s as the magnetic rotational frequency is doubled from 7.5 to 15 Hz.

The acoustic-based reaction force that is responsible for propulsion is perpendicular to the gravitational force and points in directions toward the sidewalls. For (*F*_R_)/(*F*_AS_) > 1, the acoustic radiation force dominates, which is balanced by the Stokes drag. The magnitude of the gravitation force incorporating buoyancy, can be calculated as ${F_{\rm{g}}} = 4{\rm{/}}3\pi {a^3}g\left( {{\rho _{\rm{s}}} - {\rho _0}} \right)$, where *g* = 9.81 m/s^2^ is the gravitational constant, is in the range of piconewtons. The velocities of different sized particles reaching the sidewalls of the channel ranges from 5 to 10 μm/s. Although, both the acoustic radiation and the gravitation forces scale with the particle volume, the radiation force of a 10 µm polystyrene particles is approximately two orders of magnitude larger than its gravitational counterparts.

We believe that the rolling mechanism can potentially be used for in vivo applications. The developed method is not expected to lead to thrombosis as the local shear rate developed by rolling close to the wall is unlikely to prompt any shear-induced platelet activation. The shear rate, which is estimated as $\dot \gamma = \frac{v}{h} < 1\,{\rm{per}}\,{\rm{second}}$, where *v* ~20 μm/s is the rolling speed, and *h* ~30 μm is the diameter, is of at least few orders lower than the shear-induced platelet activation^60^.

### Particle motion in 3D channel

**Fig. 6:**
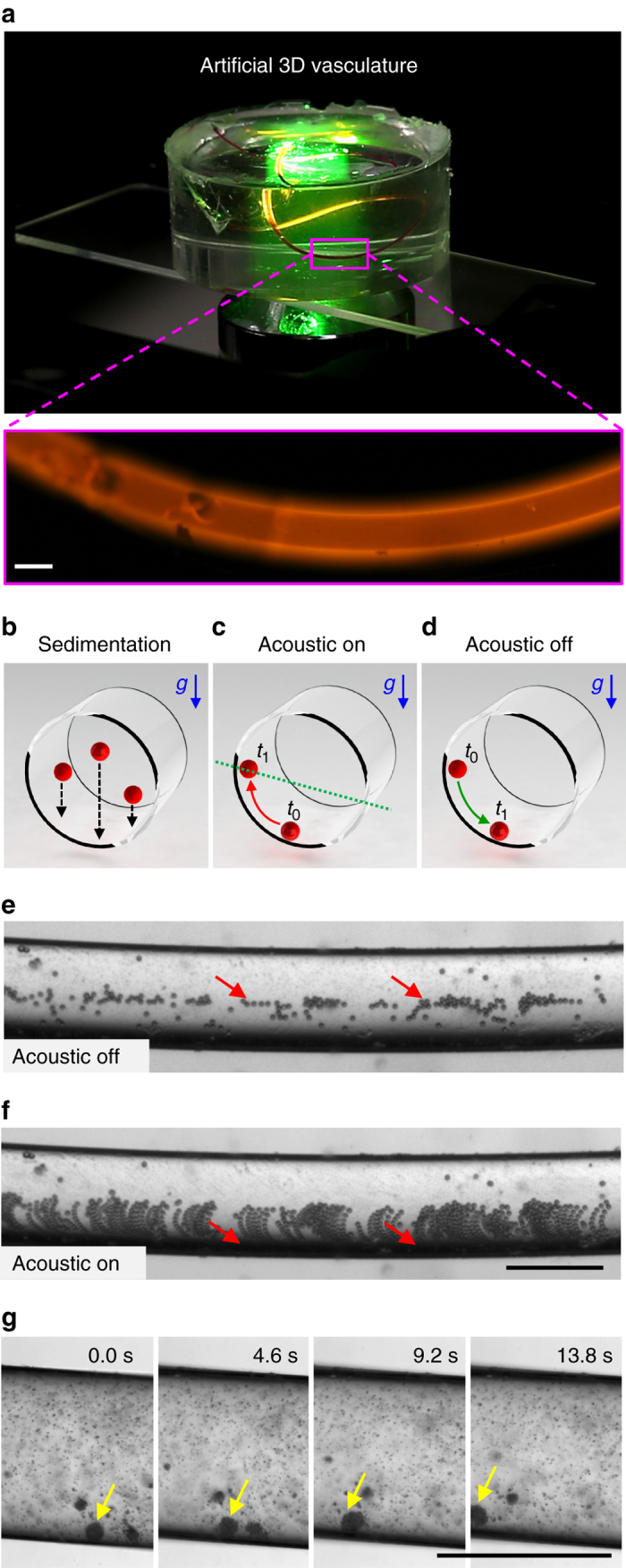
Propulsion in a circular cross-sectional artificial vasculature. **a** The image represents a three-dimensional PDMS-based artificial vasculature with a circular cross section. The *inset* shows the fluorescently labelled channel. **b** The particles sediment vertically downward towards the nearest surface and then slowly roll down to the bottom of the channel due to gravity. **c** Particles roll up along the circular channel from state *t*_0_ to *t*_1_ in the presence of acoustic waves. The *green dashed line* in **c** represents the maximum height particles can reach. **d** Particle rolls down along the surface, from state *t*_0_ to *t*_1_ in the absence of acoustic waves. **e** The micrograph shows that particles aligned at the bottom of the channel in the absence of the acoustic waves. **f** Image sequence demonstrates the particles migration is moving upwards along the channel in the presence of the acoustic field, see also Supplementary Movie 8. **g** Rolling motion is executed in a circular channel, see also Supplementary Movie 9. All *scale bars*, 500 µm

Developing propulsion with a geometric configuration that closely matches physiological settings is essential. Circular cross-sectional artificial vasculature are fabricated using a mould replica technique, as shown in Fig. 6a (see also ‘Methods’ section). The *inset* represents a channel filled with a fluorescently labelled dye. The channels have relatively large radii of curvature; as a result, imaging under microscopes with a high magnification objective becomes difficult. We used a low magnification objective lens (×2) and large particles (~1  µm in diameter) to compensate for the depth-of-field. To elucidate the acoustic trapping mechanism within the circular channel, particles are allowed to sediment at the bottom. The particles move vertically downward due to gravity and come in contact with the nearest surface (Fig. 6b). They gradually roll down along the curved surface and align themselves at the bottom of the channel. The sedimentation process is not required as particles interact with the boundary in an acoustic field. However, imaging and characterisation of particle migration become difficult as particles are trapped at different depths along the circular vessel.

When the acoustic field is activated, particles roll up along the circumference against the gravitational and contact forces from configuration *t*_0_ to *t*_1_, as shown in Fig. 6c. Once the acoustic field is turned off, the particles slowly roll down along the surface and realign themselves at the bottom, as shown in Fig. 6d. Figure 6e demonstrates sedimentation and alignment in the absence of an acoustic field. As soon as the acoustic field is turned on, the particles roll up along the surface. Figure 6f shows the image sequence of particle trajectories along the curved channel (see also Supplementary Movie 8). To verify our rolling-based propulsion along an artificial vasculature, superparamagnetic particles are injected into the channel. A rotating magnetic field is used to assemble particles above the critical diameter *a*_c_. When an acoustic field is applied, particles migrate to the channel wall, and a subsequent rolling motion results along the vessel, as shown in Fig. 6g (see also Supplementary Movie 9).

## Discussion

We demonstrated a mechanism that self-assembles single particles and then transports them along boundaries of the microchannels by a combined acoustic and magnetic actuation technique. Our mechanism provides a potential design strategy to deliver drugs in hard-to-reach capillaries and can become advantageous in delivering nanodrugs to a tumour or leaky vasculature. Nanodrugs rarely reaches the tumour site due to factors arising from the interstitial fluid pressure, the slow diffusion rate of particles in the tissue, impaired blood supply, etc^61, 62^. We look forward to functionalizing nanodrugs with the iron-oxide particles to allow mapping of the vasculature and a simultaneous transportation.

The self-assembly of our swimmers also has numerous advantages over already synthesised or fabricated microswimmers, which have limited biocompatibility, i.e. they require additional surface modifications, which can be challenging for in vivo applications. In contrast, we can use biocompatible magnetic particles already approved for use as contrast agents for magnetic resonance imaging. In addition, synthesised swimmers are associated with sophisticated and expensive fabrication procedures, whereas our concept involves a simple method of operation using commercially available superparamagnetic particles.

Currently, we have demonstrated rolling motion under no flow, and we assume that being close to the boundary may become favourable for the motion against the flow. The assembled superparamagnetic particles close to a wall experience many forces and torques that could become beneficial for particle margination and transport along or against the flow. As a proof-of-concept, we demonstrated assembly of superparamagnetic particles under a flow in a magnetic field (see also Supplementary Fig. 6 and Supplementary Movie 10). Future work will include a thorough investigation of rolling behaviour at different flow conditions.

## Methods

### Experimental setup

Our experimental design includes PDMS-based rectangular and circular cross-sectional microchannels and piezoelectric transducers (see also Supplementary Fig. 1). The transducer component is mounted on a glass slide adjacent to the channel using a thin layer of two-component epoxy glue, and is connected to an electronic function generator. The setup is mounted on an inverted microscope (Olympus IX 81) and imaged through a sensitive CMOS camera. The excitation frequency of the acoustic waves is modulated from 20 to 200 kHz, at 10 to 30 V_PP_. A custom built electromagnetic system (MagnebotiX MFG-100-i) with eight independently controlled coils was integrated with the microscope to generate the magnetic fields in the system, see also Supplementary Fig. 2. A maximum field strength and rotation frequency of 20 mT and 22.5 Hz, respectively, were used in our experiments. Commercially available superparamagnetic particles of diameter 1–2 µm (Polysciences) and 2.9 µm (COMPEL, Bangs Laboratories) are used in the experiments. A combination of acoustic and magnetic fields was utilised to demonstrate rolling motions near boundaries or walls.

### Microfluidic channel

A straight single-layer PDMS microchannel was fabricated using a conventional soft lithography and mould replica technique. A silicon master mould for the microchannel was patterned using a positive photoresist (Shipley 1827, MicroChem, USA) and etched. The mould was then vapour coated with 1H,1H,2H,2H-perfluorooctyltrichlorosilane (Sigma Aldrich, USA) such that the master mould does not disintegrate and to avoid any subsequent damage to the PDMS channel during the demoulding process. Sylgard 184 Silicone Elastomer Base and Sylgard 184 Silicone Elastomer Curing Agent (Dow Corning, USA) were mixed at a 10:1 (weight:weight) ratio and cast onto the silicon mould. The uncured PDMS on the silicon mould was then degassed in a vacuum desiccator for 2 h to remove any air microbubbles and later cured at 65 °C for 2 h. After gently removing the cured PDMS from the silicon mould, the inlets and the outlets were punched into the PDMS using a reusable biopsy punch (Harris Uni-Core, Ted Pella, USA). Then, the PDMS device was bonded to the cover glass after plasma treatment.

### 3D PDMS-based circular channel

3D channels are made by moulding electric wires into PDMS, which are removed after curing. Electrical wires of diameter 500 μm are embedded in a six-well plate (Corning). Then a 10:1 PDMS mixture is poured into the wells. To remove gas bubbles, they are refrigerated at −4 °C. The wells are kept at room temperature for half an hour and then placed on a hot plate at 60 °C for an hour. The wires are then carefully pulled out from the cured PDMS gels.

### Data availability

The authors declare that data supporting the findings of this study are available within the paper and its supplementary information files.

## Electronic supplementary material

Supplementary Information

Description of Additional Supplementary Files

Supplementary Movie 1

Supplementary Movie 2

Supplementary Movie 3

Supplementary Movie 4

Supplementary Movie 5

Supplementary Movie 6

Supplementary Movie 7

Supplementary Movie 8

Supplementary Movie 9

Supplementary Movie 10

## References

[1] Purcell EM (1977). Life at low Reynolds number. Am. J. Phys..

[2] Berg HC (1975). Bacterial behaviour. Nature.

[3] Gray J, Hancock GJ (1955). The propulsion of sea-urchin spermatozoa. J. Exp. Biol..

[4] Nosrati R, Driouchi A, Yip CM, Sinton D (2015). Two-dimensional slither swimming of sperm within a micrometre of a surface. Nat. Commun..

[5] Drescher K (2009). Dancing volvox: hydrodynamic bound states of swimming algae. Phys. Rev. Lett..

[6] Williams BJ, Anand SV, Rajagopalan J, Saif MTA (2014). A self-propelled biohybrid swimmer at low Reynolds number. Nat. Commun..

[7] Sanchez S, Soler L, Katuri J (2015). Chemically powered micro- and nanomotors. Angew Chem. Int. Ed..

[8] Soler L, Magdanz V, Fomin VM, Sanchez S, Schmidt OG (2013). Self-propelled micromotors for cleaning polluted water. ACS Nano.

[9] Soler L, Sanchez S (2014). Catalytic nanomotors for environmental monitoring and water remediation. Nanoscale.

[10] Srivastava SK, Guix M, Schmidt OG (2015). Wastewater mediated activation of micromotors for efficient water cleaning. Nano Lett..

[11] Teo WZ, Pumera M (2016). Motion control of micro-/nanomotors. Chem. A Eur. J..

[12] Wang H, Pumera M (2015). Fabrication of micro/nanoscale motors. Chem. Rev..

[13] Pumera M (2010). Electrochemically powered self-propelled electrophoretic nanosubmarines. Nanoscale.

[14] Kherzi B, Pumera M (2016). Self-propelled autonomous nanomotors meet microfluidics. Nanoscale.

[15] Chang ST, Paunov VN, Petsev DN, Velev OD (2007). Remotely powered self-propelling particles and micropumps based on miniature diodes. Nat. Mater..

[16] Loget G, Kuhn A (2011). Electric field-induced chemical locomotion of conducting objects. Nat. Commun..

[17] Palagi S (2016). Structured light enables biomimetic swimming and versatile locomotion of photoresponsive soft microrobots. Nat. Mater..

[18] Liu M, Zentgraf T, Liu Y, Bartal G, Zhang X (2010). Light-driven nanoscale plasmonic motors. Nat. Nanotechnol..

[19] Dai B (2016). Programmable artificial phototactic microswimmer. Nat. Nanotechnol..

[20] Tasci TO, Herson PS, Neeves KB, Marr DWM (2016). Surface-enabled propulsion and control of colloidal microwheels. Nat. Commun..

[21] Jang B (2015). Undulatory locomotion of magnetic multilink nanoswimmers. Nano Lett..

[22] Huang H-W, Sakar MS, Petruska AJ, Pane S, Nelson BJ (2016). Soft micromachines with programmable motility and morphology. Nat. Commun..

[23] Nelson BJ, Kaliakatsos IK, Abbott JJ (2010). Microrobots for minimally invasive medicine. Annu. Rev. Biomed. Eng..

[24] Klotsa D, Baldwin KA, Hill RJA, Bowley RM, Swift MR (2015). Propulsion of a two-sphere swimmer. Phys. Rev. Lett..

[25] Ahmed D (2017). Artificial swimmers propelled by acoustically activated flagella. Nano Lett..

[26] Dijkink RJ, Dennen JPVan, Der, Ohl CD (2006). & Prosperetti, a. The ‘acoustic scallop’: a bubble-powered actuator. J. Micromech. Microeng..

[27] Ahmed D (2015). Selectively manipulable acoustic-powered microswimmers. Sci. Rep..

[28] Wang W, Castro LA, Hoyos M, Mallouk TE (2012). Autonomous motion of metallic microrods propelled by ultrasound. ACS Nano.

[29] Bertin N (2015). Propulsion of bubble-based acoustic microswimmers. Phys. Rev. Appl..

[30] Li J (2015). Magneto-acoustic hybrid nanomotor. Nano Lett..

[31] Ahmed D, Dillinger C, Hong A, Nelson BJ (2017). Artificial acousto-magnetic soft microswimmers. Adv. Mater. Technol..

[32] Ahmed D, Mao X, Shi J, Juluri BK, Huang TJ (2009). A millisecond micromixer via single-bubble-based acoustic streaming. Lab. Chip.

[33] Ahmed D, Mao X, Juluri BK, Huang TJ (2009). A fast microfluidic mixer based on acoustically driven sidewall-trapped microbubbles. Microfluid. Nanofluid..

[34] Ozcelik A (2014). An acousto fluidic micromixer via bubble inception and cavitation from microchannel sidewalls. Anal. Chem..

[35] Ahmed D (2014). Acousto fluidic chemical waveform generator and switch. Anal. Chem..

[36] Ahmed D (2013). Tunable, pulsatile chemical gradient generation via acoustically driven oscillating bubbles. Lab. Chip.

[37] Shi J (2009). Acoustic tweezers: patterning cells and microparticles using standing surface acoustic waves (SSAW). Lab. Chip.

[38] Collins DJ (2015). Two-dimensional single-cell patterning with one cell per well driven by surface acoustic waves. Nat. Commun..

[39] Xie Y (2014). Acoustofluidic relay: sequential trapping and transporting of microparticles via acoustically excited oscillating bubbles. J. Lab. Autom..

[40] Schwarz T, Hahn P, Petit-Pierre G, Dual J (2014). Rotation of fibers and other non-spherical particles by the acoustic radiation torque. Microfluid. Nanofluid..

[41] Marzo A (2015). Holographic acoustic elements for manipulation of levitated objects. Nat. Commun..

[42] Ahmed D (2016). Rotational manipulation of single cells and organisms using acoustic waves. Nat. Commun..

[43] Zhou Q, Sariola V, Latifi K, Liimatainen V (2016). Controlling the motion of multiple objects on a Chladni plate. Nat. Commun..

[44] Wiklund M (2012). Acoustofluidics 12: biocompatibility and cell viability in microfluidic acoustic resonators. Lab. Chip.

[45] Vanherberghen B (2010). Ultrasound-controlled cell aggregation in a multi-well chip. Lab. Chip.

[46] Ding X (2014). Cell separation using tilted-angle standing surface acoustic waves. Proc. Natl Acad. Sci. USA.

[47] Chen K (2016). Rapid formation of size-controllable multicellular spheroids via 3D acoustic tweezers. Lab. Chip.

[48] Errico C (2015). Ultrafast ultrasound localization microscopy for deep super-resolution vascular imaging. Nature.

[49] Christensen-Jeffries K, Browning RJ, Tang MX, Dunsby C, Eckersley RJ (2015). In vivo acoustic super-resolution and super-resolved velocity mapping using microbubbles. IEEE Trans. Med. Imaging.

[50] Carpentier A (2016). Clinical trial of blood-brain barrier disruption by pulsed ultrasound. Sci. Transl. Med..

[51] Schoellhammer CM (2015). Ultrasound-mediated gastrointestinal drug delivery. Sci. Transl. Med..

[52] Bremond N, Arora M, Ohl CD, Lohse D (2006). Controlled multibubble surface cavitation. Phys. Rev. Lett..

[53] Zwaan E, Le Gac S, Tsuji K, Ohl CD (2007). Controlled cavitation in microfluidic systems. Phys. Rev. Lett..

[54] Feng J, Yuan J, Cho SK (2015). Micropropulsion by an acoustic bubble for navigating microfluidic spaces. Lab. Chip.

[55] Chen H, Kreider W, Brayman AA, Bailey MR, Matula TJ (2011). Blood vessel deformations on microsecond time scales by ultrasonic cavitation. Phys. Rev. Lett..

[56] Azhari, H. *Basics of Biomedical Ultrasound For Engineers* (Wiley, 2010).

[57] Bruus H (2012). Acoustofluidics 10: scaling laws in acoustophoresis. Lab. Chip.

[58] Baasch T, Leibacher I (2017). Multibody dynamics in acoustophoresis. J. Acoust. Soc. Am..

[59] Keshoju K, Xing H, Sun L (2007). Magnetic field driven nanowire rotation in suspension. Appl. Phys. Lett..

[60] Holme PA (1997). Shear-induced platelet activation and platelet microparticle formation at blood flow conditions as in arteries with a severe stenosis. Arterioscler. Thromb. Vasc. Biol..

[61] Jain RK, Stylianopoulos T (2011). Delivering nanomedicine to solid tumors. Nat. Rev. Clin. Oncol..

[62] Nichols JW, Bae YH (2012). Odyssey of a cancer nanoparticle: from injection site to site of action. Nano Today.

