# Supplementary material for: Neutrophil-inspired propulsion in a combined acoustic and magnetic field
Source: Nat Commun. 2017 Oct 3;8:770. doi: 10.1038/s41467-017-00845-5 (PMC5626690; doi:10.1038/s41467-017-00845-5)
Supplement: Supplementary file 1 — Supplementary Information [file 41467_2017_845_MOESM1_ESM.pdf]

Supplementary Figures and Tables

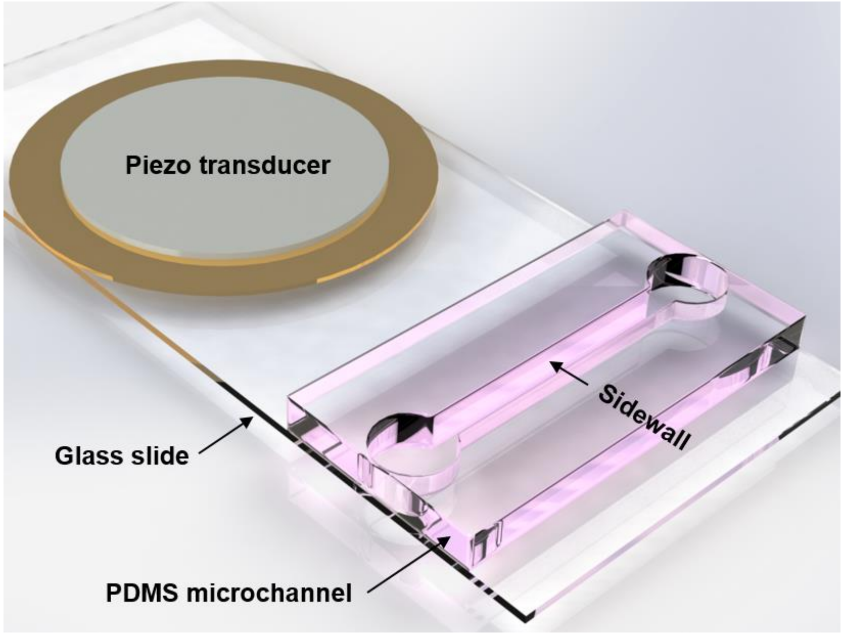

Supplementary Figure 1| Acoustic Setup. The transducer component is mounted on a glass slide adjacent to the microfluidic channel using a thin layer of a two-component epoxy glue, and is connected to an electronic function generator. The setup is mounted on an inverted microscope (Olympus IX 81) and imaged through CMOS camera. The excitation frequency of the acoustic waves is modulated from 60 to $200\ \mathrm{kHz}$, at 10 -30 V_{PP}

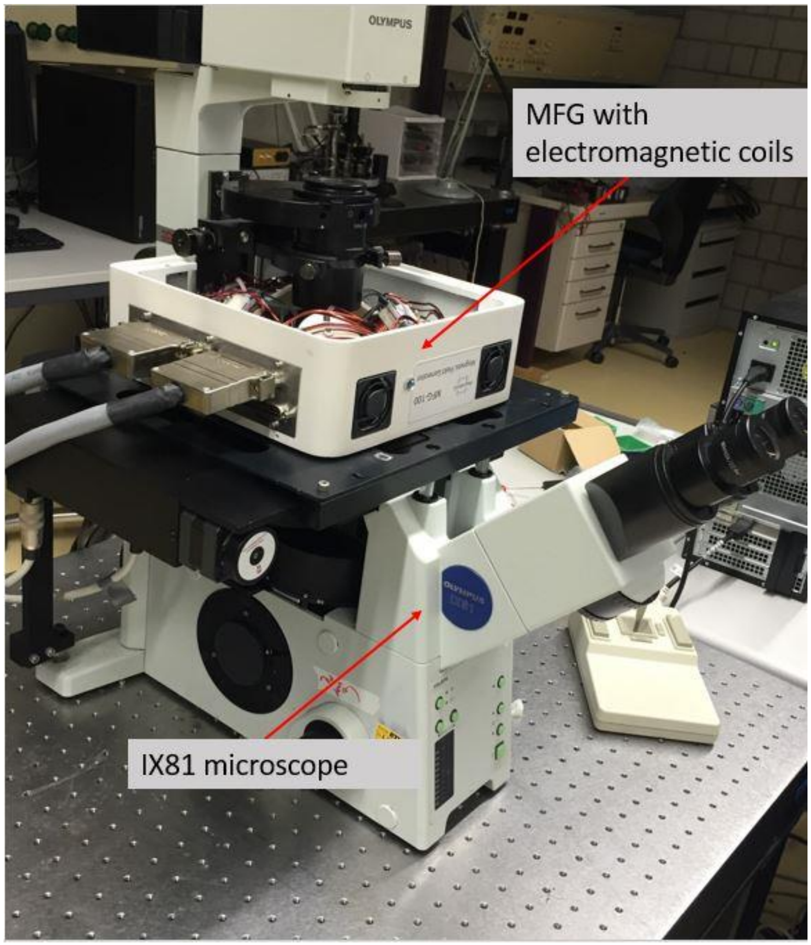

Supplementary Figure 2| Magnetic setup. A custom built electromagnetic system (MagnebotiX MFG-100-i) with eight independently controlled coils was integrated with the Olympus IX 81 inverted microscope to generate a rotating magnetic field.

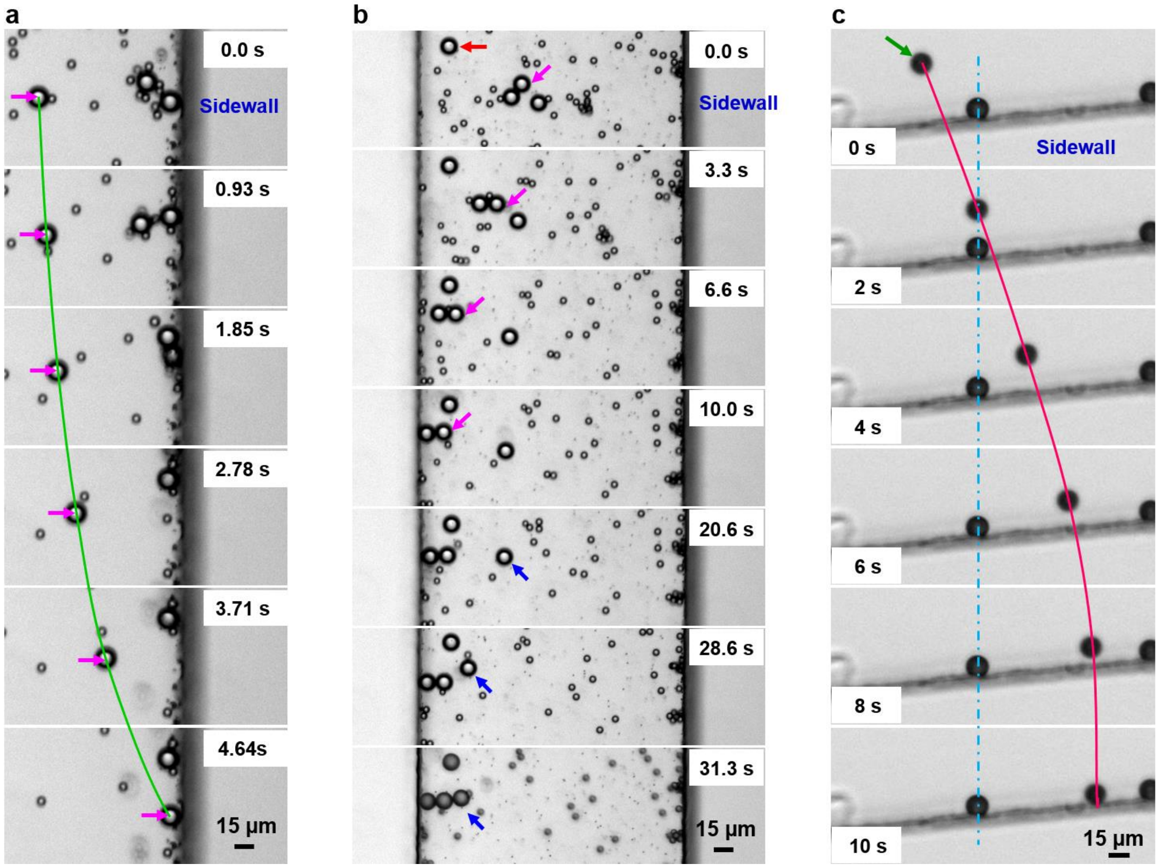

Supplementary Figure 3| Interparticle interaction near the channel sidewalls. (a) Image sequence demonstrates a polystyrene particle moves towards the sidewall at a uniform velocity, however, as it approaches the wall it accelerates, as indicated in the last frame. (b) Image sequence demonstrates the chain formation at the wall. (c) Image sequence exhibits a sudden change in the particle trajectory as it approaches the sidewall.

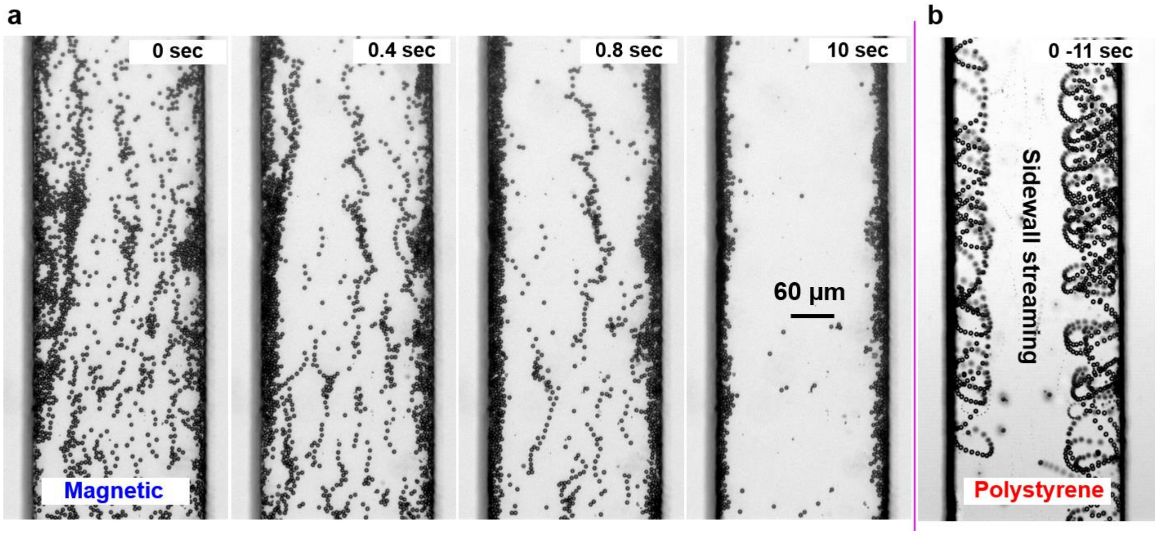

Supplementary Figure 4| Particle-wall interaction of superparamagnetic and polystyrene particles of similar diameter. (a) Image-sequence demonstrates the superparamagnetic particles migrate to the sidewalls of the microchannel. (b) Image sequence illustrates the polystyrene particles undergo sidewall-streaming (see also Supplementary Movie 2).

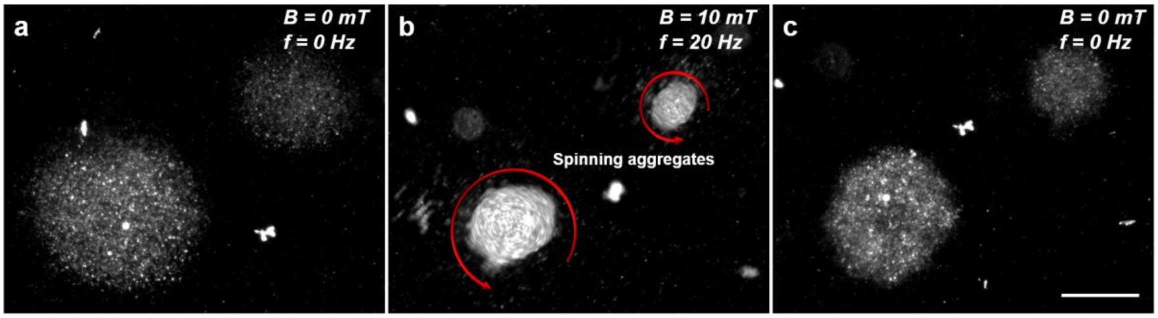

Supplementary Figure 5| Assembly and disassembly of 1-2 superparamagnetic particles under the influence of an external rotating magnetic field (see also **Supplementary Movie 5**). (a) Particles remain stationary in the absence of magnetic field. (b) Particles self-assemble in the presence of rotating magnetic field of 20 Hz at 10 mT. Note the assembly condenses under an external magnetic field. (c) The particles disassemble once the magnetic field is turned off and show no magnetic remanence.

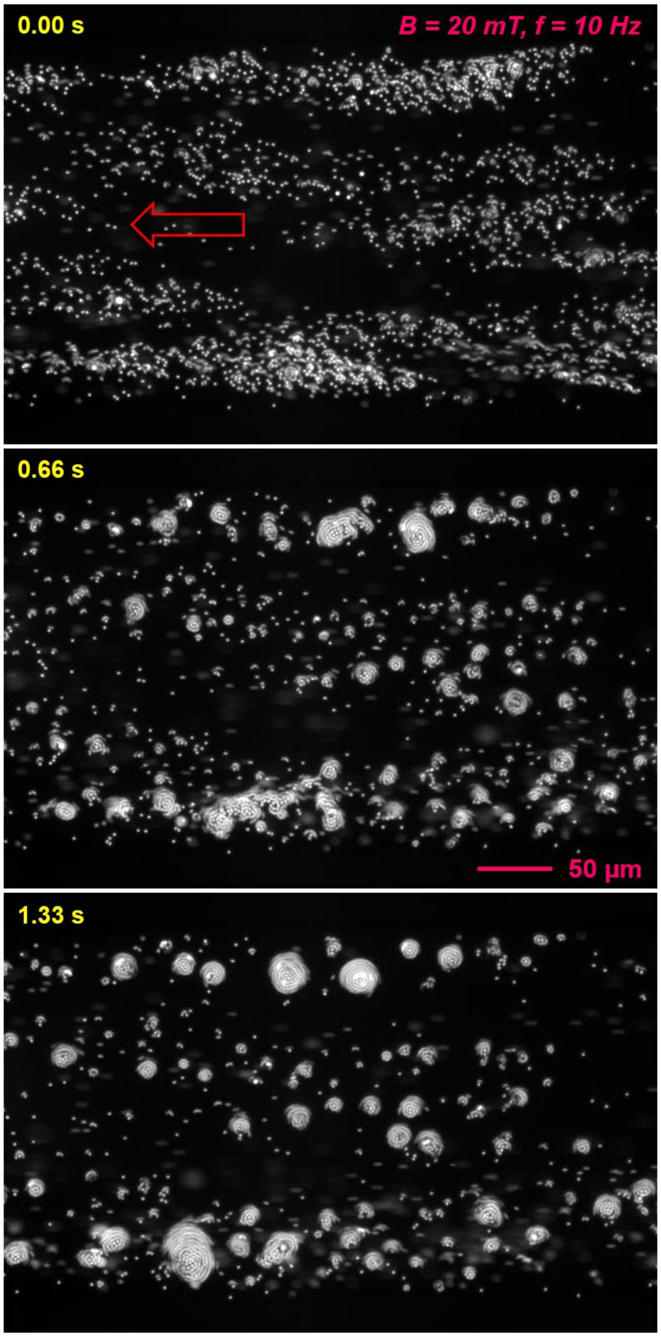

Supplementary Figure 6| The Assembly of 2.9 µm superparamagnetic particles in a flow under an external rotating magnetic field of 20 mT and 10 Hz, (**see also Supplementary Movie 10)**. The solutions containing magnetic particles are manually injected into the microfluidic channel. Due to a pressure difference between the inlet and the outlet of the microchannel, a laminar flow is established. When the rotating magnetic field is applied, the particles begin to assemble. For an initial flow velocity of ~ 0.2 mm/s, particle assembles in seconds.

Material properties	Symbol	Value
Water		
• Density	ρ_0	1000 kg·m^{-3}
• Speed of sound	c_0	1500 m·s^{-1}
Polystyrene particles		
• Density	ρ_{PS}	1050 kg·m^{-3}
• Speed of sound	c_{PS}	2350 m·s^{-1}
Polystyrene- 20% iron particles		
• Composite density*	$\rho_{\mathrm{PS/Fe}}$	2000 kg·m^{-3}
• Speed of sound in iron	c_{Fe}	3000 m·s^{-1}
• Speed of sound in polystyrene	c_{PS}	2350 m·s^{-1}

***** Provided in the technical data sheet by the manufacturer (Polysciences Inc.).

Supplementary Table 1| Material properties used in the experiments.

Supplementary Note 1: The acoustic contrast factor of superparamagnetic particles

The 1-2 superparamagnetic particles from Polysciences Inc. are composed of 20% iron particles in the polymer material. The acoustic contrast factor ϕ can be calculated by the expression below[1]

$$\phi = \frac{f_1}{3} + \frac{f_2}{2} \quad (1)$$

where,

$$f_1 = 1 - \frac{\kappa_{\mathrm{PS/Fe}}}{\kappa_0} \quad (2)$$

and,

$$f_2 = \frac{2(\rho_{\mathrm{PS/Fe}} - \rho_0)}{2\rho_{\mathrm{PS/Fe}} + \rho_0} \quad (3)$$

The combined density, $\rho_{\mathrm{PS/Fe}}$ of the superparamagnetic particle is provided in the technical data sheet by the manufacturer (Polysciences Inc.) and is 2000 $\mathrm{kg \cdot m^{-3}}$. The combined compressibility of the polymer/iron composite, $\kappa_{\mathrm{PS/Fe}}$, can be obtained by the following expression[1]

$$\kappa_{\mathrm{PS/Fe}} = \frac{1}{V}(V_{\mathrm{Fe}}\kappa_{\mathrm{Fe}} + V_{\mathrm{PS}}\kappa_{\mathrm{PS}}) \quad (4)$$

Where, $V_{\mathrm{Fe}}, V_{\mathrm{PS}}$ is the volume fraction of iron particles and the polymer, respectively and $\kappa_{\mathrm{Fe}}, \kappa_{\mathrm{PS}}$ is the compressibility of the iron and polymer, respectively.

Supplementary Note 2: Simulation (COMSOL) Setup

We simulated the device setup using Comsol Multiphysics, which provides a qualitative understanding of the particle trapping behaviour towards the sidewall. The geometry of the device was modelled as two-dimensional due to computational limitations. The piezoelectric transducer was modelled using the material properties of PZ26. The material properties of the PDMS, water, and glass slide can be found in the table shown below. The deformations of the glass slide were modelled using the structural mechanics module, which includes both the primary and secondary waves. The piezoelectric transducer was modelled by coupling the structural mechanics module with the piezoelectricity. The acoustic domain includes both the PDMS and the water filled channel. In this domain, the secondary waves were neglected, and all the domains were coupled at the interfaces.

The simulations show that there is a significant vibration in the glass slide, which induces a pressure wave in the water channel. The gradient of the Gor'kov potential indicates that particles will be attracted to the bottom of the microchannel and, depending on the excitation frequency, will be driven to one of the sidewalls.

	Density (kg/m^3)	Speed of sound (m/s)	Young's modulus (Pa)	Poisson ratio (-)
Water	1000	1500		
PDMS	1000	1000*(1+i*1/200)		
Glass	2240		6E10*(1+i/2420)	0.245

Supplementary Table 2| Materials properties used in the COMSOL simulation.

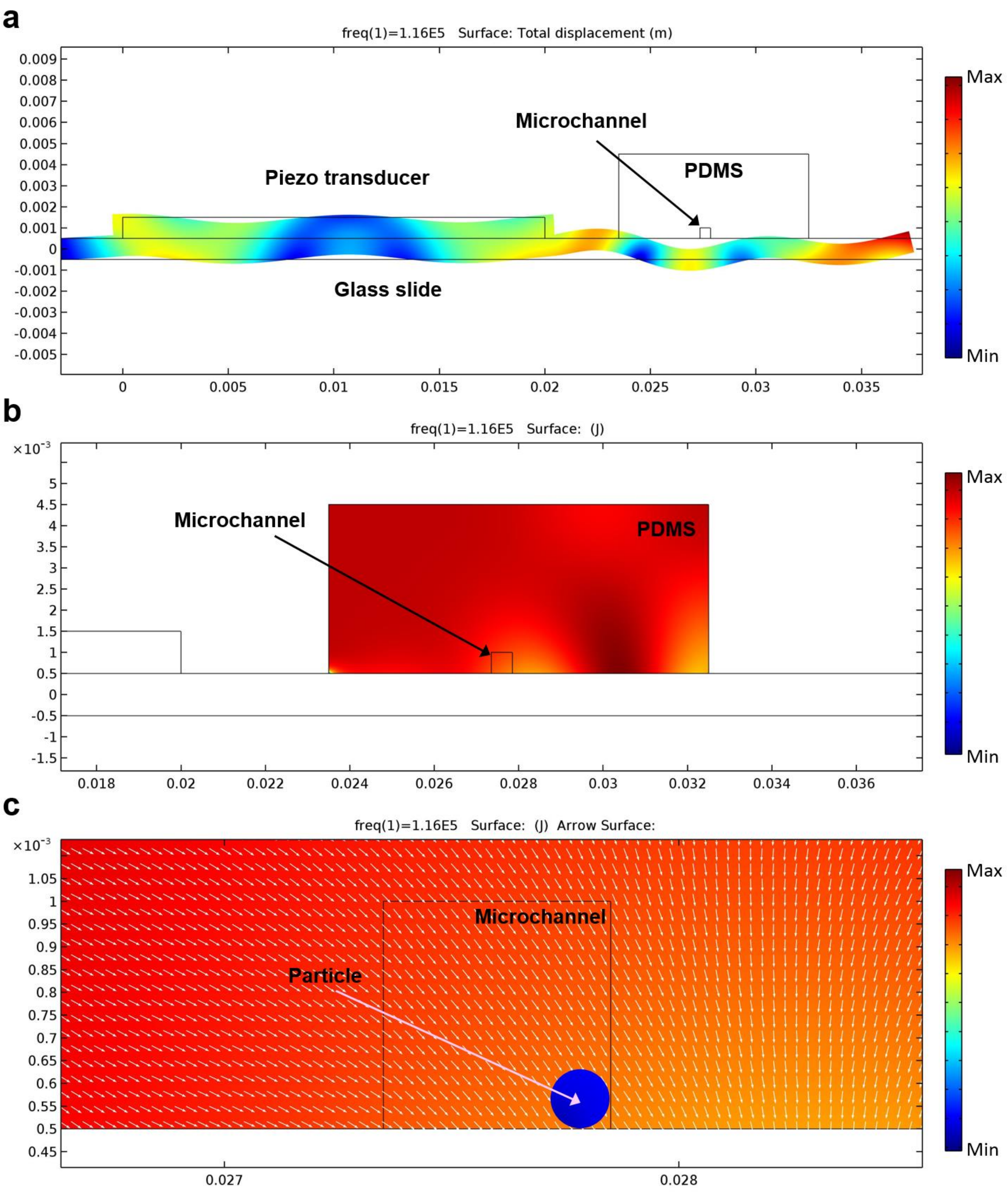

Supplementary Figure 7| COMSOL simulations demonstrate (a) setup vibration, (b) Gor’kov Potential, and (c) the stable position of the particle, which is at the boundary of the microchannel. White arrows show the gradient of the potential for positive acoustic contrast factor ϕ.

Supplementary References

1. Leibacher, I., Dietze, W. & Hahn, P. Acoustophoresis of hollow and core-shell particles in two-dimensional resonance modes. *Microfluid. Nanofluidics* **16,** 513–524 (2014).
