# Supplementary material for: Neutrophil-inspired propulsion in a combined acoustic and magnetic field
Source: Nat Commun. 2017 Oct 3;8:770. doi: 10.1038/s41467-017-00845-5 (PMC5626690; doi:10.1038/s41467-017-00845-5)
Supplement: Supplementary file 2 — Description of Additional Supplementary Files [file 41467_2017_845_MOESM2_ESM.pdf]

Description of Additional Supplementary Files

File Name: Supplementary Movie 1
Description: Comparison of polystyrene particle-wall interaction in an acoustic field of 3.0, 6.0, 10.0 and 15.5 µm diameter particles, respectively, in microfluidic channels. The video is captured at 15 fps and is played at 45 fps.

File Name: Supplementary Movie 2
Description: Comparison of particle-wall interaction of ~ 6.0 µm polystyrene and magnetic particles in an acoustic field. The video is captured at 15 fps and is played at 45 fps.

File Name: Supplementary Movie 3
Description: Transformation of fluorescent particles from spinning chains to Neutrophil-like aggregates by tuning the magnetic field frequency from 0.5 to 20 Hz at 10 mT. The video is captured at ~15 fps and played at 30 fps.

File Name: Supplementary Movie 4
Description: Propulsion of spinning aggregates in combination of acoustic and magnetic field along the sidewall of a microfluidic channel. The video is captured at ~15 fps and played at 30 fps.

File Name: Supplementary Movie 5
Description: Assembly and disassembly of the spinning aggregates. The video is captured at ~15 fps and played at 30 fps.

File Name: Supplementary Movie 6
Description: Counter-clockwise motion of an aggregate and executing rolling when reached the wall under a magnetic field strength and rotation frequency of 20 mT and 20 Hz, respectively at an acoustic driving voltage of 20 VPP. The video is captured at 15 fps and played at 30 fps.

File Name: Supplementary Movie 7
Description: An aggregate is shown weak to no rolling at low acoustic driving voltages of 10 VPP under a magnetic field strength and rotation frequency of 15 mT and 7.5 Hz. The video is captured at 15 fps and played 15 fps.

File Name: Supplementary Movie 8
Description: Particle-wall interaction in a three-dimensional artificial vasculature in an acoustic field. The movie is captured at 15 fps (stored by skipping every 2 frames) and is played at 120 fps.

File Name: Supplementary Movie 9
Description: Rolling motion of assembled 2.9 µm superparamagnetic particles at a magnetic field strength and the rotation frequency of 15 mT and 15 Hz, respectively, at an acoustic driving voltage of 30 VP. The video is captured at 15 fps and played at 15 fps.

File Name: Supplementary Movie 10
Description: The assembly of 2.9 µm superparamagnetic particles in a flow at a magnetic field strength and the rotation frequency of 20 mT and 10 Hz, respectively. The video is captured at 30 fps and played at 30 fps
